# A Multi-Module Electrodynamic Exciter with a Variable Pole-Arc Ratio Disk Halbach Array for a High-Bandwidth Dynamic Torsional Stiffness Test

**DOI:** 10.3390/s19061272

**Published:** 2019-03-13

**Authors:** Fang Yuan, Lizhan Zeng, Xuedong Chen, Chengyuan Liang, Xiaojun Ding, Xin Luo

**Affiliations:** State Key Laboratory of Digital Manufacturing Equipment and Technology, Huazhong University of Science and Technology, Wuhan 430074, China; bigsquare@hust.edu.cn (F.Y.); zenglizhan@hust.edu.cn (L.Z.); chenxd@hust.edu.cn (X.C.); D201177131@hust.edu.cn (C.L.); ding_xj@hust.edu.cn (X.D.)

**Keywords:** dynamic torsional stiffness test, electrodynamic exciter, disk voice coil motor, Halbach array, variable pole-arc ratio, quasi-3-D model

## Abstract

In this paper, a multi-module electrodynamic exciter based on moving-magnet disk voice coil motor is presented to meet the demands of high torque and high bandwidth in a dynamic torsional stiffness test. A variable pole-arc ratio disk Halbach array (VPAR-DHA) is proposed, so that both high torque density and low rotor inertia can be obtained through enhancing the magnetic field in the working range. The analytical quasi-3-D model of VPAR-DHA was set up by using the harmonic function method, with the consideration of end-effects by a correction function. Electromagnetic structure optimization was carried out with the analytical model, and verified by 3-D finite-element (FEM) results. The proposed design was experimentally tested and verified with a prototype that achieved a peak dynamic torque output of 40 Nm at a frequency of 120 Hz, and a stroke of ±1°. The proposed method can also be easily extended to satisfy various demands of dynamic torsional stiffness test.

## 1. Introduction

Stiffness is an important evaluation index for mechanical structures, which describes the extent to which an object resists deformation in response to an applied force. For a rotating machinery system, the output dynamic stiffness reflects the resistant ability of the structure of a system against dynamic torsional disturbance. A working knowledge of dynamic stiffness for the machinery engineer is as useful and indispensable as Ohm’s Law is for the electrical engineer. The dynamic torsional stiffness test for rotating structures has been extensively studied [1,2,3,4], in which a dynamic exciter plays a key role by providing dynamic torque in a short-range reciprocating motion [5]. With the growing demand of high-frequency actuation in applications such as motor vehicles and aerospace equipment [4], the design of the dynamic exciter has been confronted with the contradiction between high torque and high frequency, since the acceleration motion consumes a lot of output torque.

Electrodynamic exciters can directly provide high-dynamic precision short-stroke reciprocating movements, combined with low power losses without any motion conversion mechanisms [6], and it has a faster dynamic response than other kinds of dynamic exciters, such as hydraulic ones. With the improvement of modern permanent magnet synchronous motor (PMSM) torque output capabilities, electrodynamic exciters have received great attention recently, and they are widely used in dynamic torsional stiffness tests [4,5]. Compared with moving iron and moving coil motors, the moving-magnet PMSM is characterized by a smaller air gap and a higher torque density, and consequently, it has become the most suitable and common choice for electrodynamic exciters [7,8,9,10,11].

From the viewpoint of motor design, the gap radius (GR) and the axial length of the PMSM are the critical parameters that affect acceleration capability (torque per inertia). The torque is proportional to GR^2^, and the rotor inertia is proportional to GR^3^; therefore, the acceleration capability is proportional to 1/GR. Meanwhile, the axial length of the motor is proportional to the torque, and it does not affect the torque per inertia. Thus, to achieve satisfactory dynamic performance by the electrodynamic exciters, the most effective way is to improve torque per inertia by appropriately reducing the gap radius, and increasing the axial length. Compared with conventional PMSMs, disk-type permanent magnet (PM) motors can be designed in a multistage configuration with extreme axial compactness [7]; thus, they are more suitable for high-acceleration applications such as electrodynamic exciters [8,9,10]. 

In conventional disk motors, rotor magnet arrays usually introduce axial North-South alternate magnetization arrays with an inevitably thick back-iron to eliminate saturation and to create flux paths, which becomes a bottleneck for improving motor dynamic performance [7,8,9,10,11]. With the help of a Halbach array, which has a high PM working point and self-shielding magnetization, the thickness of the back-iron can be reduced or even removed, reducing the rotor inertia significantly [11]. As a result, multipole Halbach magnetized movers/rotors have been developed for various applications [12], and researchers are focused on changing the magnet shape in the Halbach array, such as a T-shape, triangle, trapezoidal shape, or a compound dual-layer structure, to increase self-shielding effects, improve the thrust/torque output, and to decrease the thrust/torque variation of the PMSMs [11,12,13,14,15]. However, the fabrication of complex magnet shapes is too difficult, especially for disk motors, which vary in structure size with radius. In general, research on the Halbach array for disk motors used in electrodynamic exciters for the dynamic stiffness test is insufficient.

This paper proposes a novel multi-module moving-magnet disk voice coil motor-based electrodynamic exciter (DVCM-EDE) for the high-bandwidth dynamic torsional stiffness test. A simple structure of the Halbach array that has a variable pole-arc ratio in radial direction was introduced for enhancing the magnetic field in the working range, and the back-iron could be removed completely to reduce the rotor inertia significantly without affecting the torque-generating ability too much. Section 2 gives the principles and structures of the proposed variable pole-arc ratio disk Halbach array (VPAR-DHA) and DVCM-EDE. Based on the VPAR-DHA structure, quasi-3-D models of the magnetic field and the torque were derived by using the harmonic function method in Section 3.1, and then the correction function of the quasi-3-D model with end-effects was considered in Section 3.2. Section 3.3 gives the electromagnetic structure optimization results based on the quasi-3-D model with 3-D finite-element method (FEM) validation. Section 4 provides the prototype design and experimental results on the static electromagnetic performance and dynamic response. Finally, a conclusion is made in Section 5.

## 2. Principle and Structure

### 2.1. Principle of the Dynamic Torsional Stiffness Test

**Figure 1:**
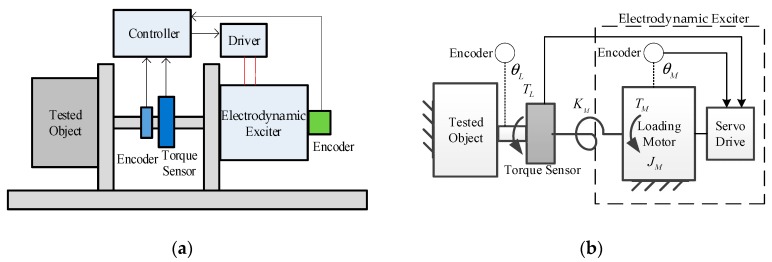
Dynamic torsional stiffness test with an electrodynamic exciter: (**a**) Principle; (**b**) Simplified diagram.

The principle of the dynamic torsional stiffness test with an electrodynamic exciter and its simplified diagram is shown in Figure 1.

The testing system mainly consists of the electrodynamic exciter, exciter driver and controller, encoders for feedback reference and for testing, torque sensor, transmission link, tested object, and mounting bases.

During testing, the tested object is locked into a specific working position. The exciter output sweeping torque signals within the loading bandwidth at the output terminal of the tested object. The Encoder and the torque sensor are arranged at the terminal to acquire real-time torque and angular signals. According to the definition, the output dynamic torsional stiffness of the tested object is given by:

(1) $$z(j\omega )=\frac{T_{L}(j\omega )}{\theta_{L}(j\omega )}$$

where $\theta_{L}$ is the position of the test end; $T_{L}$ is the load torque, which is the output of loading motor within the electrodynamic exciters. Both can be directly sampled with sensors.

The system test ability is determined by the loading capacity of the exciter. Considering the moment of inertia of the exciter, and the stiffness of the transmission link, the transfer function from the electromagnetic torque and the position of the test end to the load torque can be obtained as:

(2) $$T_{L}(s)=\frac{K_{M}}{J_{M}s^{2}+K_{M}}[T_{M}(s)-J_{M}s^{2}\theta_{L}(s)]$$

where $J_{M}$ and $T_{M}$ are the rotor inertia and electromagnetic torque of the loading motor, respectively; $K_{M}$ is the equivalent transmission stiffness.

The tested object in the dynamic torsional stiffness test often contains complex transmission mechanisms, such as a reduction gearbox or worm gear. In order to obtain real measurement results, the electrodynamic exciter often operates with $\theta_{L}$ up to degree magnitudes, and it has to implement direct driving to maintain $K_{M}$ at a high enough value. As a result, the only effective way to improve the dynamic performance of the electrodynamic exciter is to promote $T_{M}$ without the geometric multiplier growth of $J_{M}$, which is the main obstacle of the loading motor design.

### 2.2. The Variable Pole-Arc Ratio Disk Halbach Array

**Figure 2:**
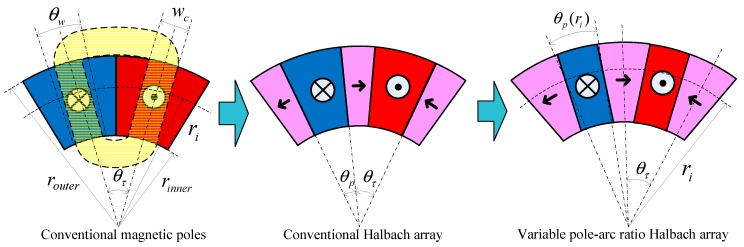
Structures of conventional and variable pole-arc ratio Halbach arrays.

To improve the output performance of the loading motor in the electrodynamic exciter, a variable pole-arc ratio disk Halbach array was introduced for DVCM rotor design, as shown in Figure 2, where $r_{outer}$ and $r_{inner}$ are the outer and inner radii of the magnet array, respectively; $r_{i}$ is the radii between $r_{outer}$ and $r_{inner}$; $\theta_{p}$ and $\theta_{\tau }$ are the mechanical angles of the axial magnetization PM and the magnetic pole, respectively; $\theta_{w}$ is the mechanical angle corresponding to the motion range of the coil; $w_{c}$ is the coil width. 

In conventional magnetic poles and conventional Halbach arrays, $\theta_{p}$ and $\theta_{\tau }$ do not vary with $r_{i}$, so that the pole-arc ratios given by $\theta_{p}/\theta_{\tau }$ are constants. To enhance the magnetic field in a range of motion, the VPAR-DHA structure was proposed based on the following facts: (1) the flux-focusing effect of the Halbach array increases with the decrease of the pole-arc ratio, while the working range of magnetic field decreases; (2) $\theta_{w}$ decreases as $r_{i}$ increases, since $w_{c}$ is a constant; (3) the outside of a coil contributes more to torque generation than the inner side, if the flux density is the same. The variable pole-arc ratio is given by:

(3) $$\alpha (r_{i})=\frac{\theta_{p}(r_{i})}{\theta_{\tau }}$$

Corresponding to $\theta_{w}$, $\theta_{p}\left(r_{i}\right)$ decreases with the increase of $r_{i}$. This structure not only ensures that the coil stays in the working range of the magnetic field, but it also enhances the average flux density $B_{av}\left(r_{i}\right)$ in the working range, with the increase of radius. As the electromagnetic torque at a certain radius $T_{M}\left(r_{i}\right)\propto B_{av}\left(r_{i}\right)$, the torque output capacity can be promoted significantly with the VPAR-DHA structure. 

### 2.3. Multi-Module Structure of the Electrodynamic Exiter

**Figure 3:**
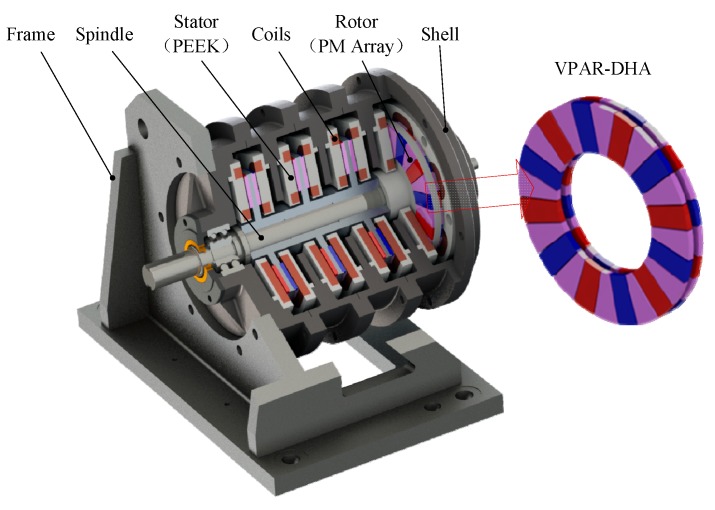
Structure of a proposed disk voice coil motor-based electrodynamic exciter (DVCM-EDE).

The torque output capability of a single DVCM is limited. To meet the various demands of the dynamic torsional stiffness test, a multi-module DVCM-EDE with VPAR-DHA structure was proposed. It is a multi-structure that contains a number of modularly designed moving-magnet-type DVCMs that provide distributed power to achieve high loads of torque-output capability, as shown in Figure 3. 

As a short-range reciprocating actuator, each DVCM was designed to be a single-phase DC-PMSM, in which the numbers of the magnetic pole and the coil are the same. Coils were assembled in polyether ether ketone (PEEK) stators, and in the stable reversing magnetic field area of the magnetic poles. The stator is ironless, and it is completely exempt from the cogging torque’s influence. As the twin stators work in reversing fields, the directions of current for each pair of stators are in the opposite directions, to generate a resultant torque. A Dual-layer VPAR-DHA pasted onto an aluminum alloy yoke works as rotor, whose inertia is remarkably reduced, with the back-iron removed. The rotors were fixed on a single spindle made of 40CrMo, to ensure the system’s stiffness. The whole structure can be characterized by direct drive, high speed, high acceleration, high positioning precision, fast dynamics, and low torque pulsation, which makes it easy to maintain.

The output capability of the exciter can be expanded by increasing the module numbers in the axial direction without reducing the torque density. As the DVCMs are modularly designed, the optimization of a single joint design will significantly improve the whole performance of the exciter. The detailed calculations and analyses are described in the following sections.

## 3. Modeling, Analysis and Optimization 

### 3.1. Analytical Modeling

**Figure 4:**
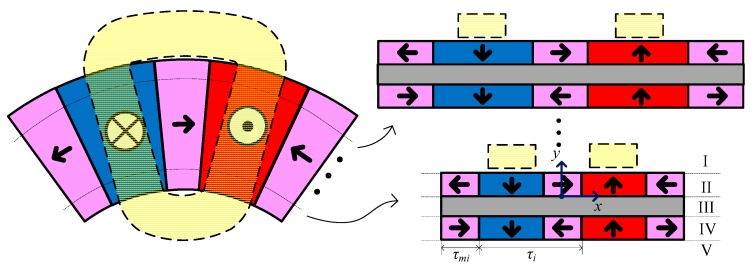
Quasi-3-D multi-slice model.

The structure size of DVCM varies with radius, so it is essential to model the magnetic field, considering its 3-D intrinsic nature, for predicting motor performances. 3-D FEM is an accurate modeling method, but it is highly time-consuming and it does not easily allow a parametric study in a design procedure. The quasi-3-D model presents a simple and effective radial-dependence modeling technique for disk motors [16,17]. According to the quasi-3-D model, as shown in Figure 4, the disk motor was divided into several linear machines in the radial direction, and each slice was modeled with a 2-D model; the performance of the disk motor can be obtained by superposition. 

In the 2-D model, the polar pitch and the magnet width vary with the radius, which can be expressed as:

(4) $$\tau_{i}=r_{i}\theta_{\tau }$$

(5) $$\tau_{mi}=[1-\alpha (r_{i})]\tau_{i}$$

As the coil in the magnetic field is subjected to the Lorentz force to produce output torque, the analytical solution for the 2-D magnetic field distribution was established, based on the following assumptions: (1) the eddy current effect and the saturation effect are ignored, (2) the permeabilities of PM and the aluminum alloy yoke are equal to that of air, (3) the end effects are neglected. Based on these assumptions, as shown in Figure 4, the 2-D model was divided into five solving regions: (I) the upper air region, (II) the upper magnet region, (III) the middle air region, representing the yoke, (IV) the lower magnet region, and (V) the lower air region. In all of these solving regions, the magnetic field satisfies the following equations:

(6) $$\begin{array}{c} B=\mu_{0}\mu_{r}H+\mu_{0}M_{0}\\ =\mu_{0}\mu_{r}H+B_{r} \end{array}$$

where $\mu_{0}$, $\mu_{r}$, $M_{0}$, and $B_{r}$ are the permeability of the vacuum, the relative permeability, the remnant magnetization, and the remanence flux density, respectively. The scalar potential can be used to solve this problem, since there is no conduction current. The governing equations can be expressed as follows:

(7) $$\left\{ \begin{array}{l} \nabla^{2}\varphi (x,y)=0\;region\;I,\;\mathrm{III},\;and\;V\\ \nabla^{2}\varphi (x,y)=\nabla \cdot M_{0}\;region\;\mathrm{II}\;and\;\mathrm{IV} \end{array}\right.$$

**Figure 5:**
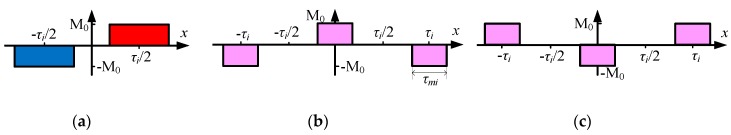
Distributions of axial and circumferential magnetization intensities: (**a**) Axial magnetized magnets of the upper and lower Halbach array. (**b**,**c**) Circumferential magnetized magnets of the upper and lower Halbach array.

The Fourier series method is utilized to derive the divergence of $M_{0}$ and to solve the flux density distribution. The upper and lower Halbach arrays were divided into two sub-arrays, namely the axial magnetized sub-array, and the circumferential magnetized sub-array, as shown in Figure 5.

The distribution of axial magnetization intensity of the upper Halbach array is the same as that of the lower one, but the distributions of circumferential magnetization intensity are in opposite directions. The distribution functions of magnetization intensity can be expressed as [18]:

(8) $$M_{1}=-\frac{B_{r}}{\mu_{0}}\sum_{k=1}^{\infty }\frac{4}{k\pi }\cos(\frac{k\pi \tau_{mi}}{2\tau_{i}})\sin(\frac{k\pi x}{\tau_{i}})$$

(9) $$M_{2}=-\frac{B_{r}}{\mu_{0}}\sum_{k=1}^{\infty }\frac{4}{k\pi }\sin(\frac{k\pi \tau_{mi}}{2\tau_{i}})\cos(\frac{k\pi x}{\tau_{i}})$$

(10) $$M_{3}=M_{1}$$

(11) $$M_{4}=-M_{2}$$

where k is the harmonic numbers, $M_{1}$ and $M_{2}$ are the Fourier series of the axial and circumferential magnetized sub-arrays of the upper Halbach array, respectively, and $M_{3}$ and $M_{4}$ are the Fourier series of the lower Halbach array.

The flux density distribution of the axial and circumferential magnetized sub-arrays of the upper Halbach array can be expressed as Equations (12) and (13), respectively, by the separation variable method [14,18] with Equations (7)–(11). Equations (14) and (15) express the flux density distribution of the lower Halbach array. In Equations (12)–(15), $H_{m}$ and $H_{y}$ are the thickness of the PM and the yoke, respectively.

(12) $$B_{1}(x,y)=\frac{-B_{r}}{2}\sum_{k=1}^{\infty }\left(e^{H_{m}k\pi /\tau_{i}}-1)e^{yk\pi /\tau_{i}}\frac{4}{k\pi }\cos(\frac{k\pi \tau_{mi}}{2\tau_{i}})\sin(\frac{k\pi x}{\tau_{i}}\right)$$

(13) $$B_{2}(x,y)=\frac{-B_{r}}{2}\sum_{k=1}^{\infty }\left(e^{H_{m}k\pi /\tau_{i}}-1)e^{yk\pi /\tau_{i}}\frac{4}{k\pi }\sin(\frac{k\pi \tau_{mi}}{2\tau_{i}})\sin(\frac{k\pi x}{\tau_{i}}\right)$$

(14) $$B_{3}(x,y)=B_{1}(x,y+H_{m}+H_{y})$$

(15) $$B_{4}(x,y)=-B_{2}(x,y+H_{m}+H_{y})$$

Then, the flux density distribution of the total magnet array can be obtained as:

(16) $$B(x,y)=\sum_{j=1}^{4}B_{j}(x,y)$$

**Figure 6:**
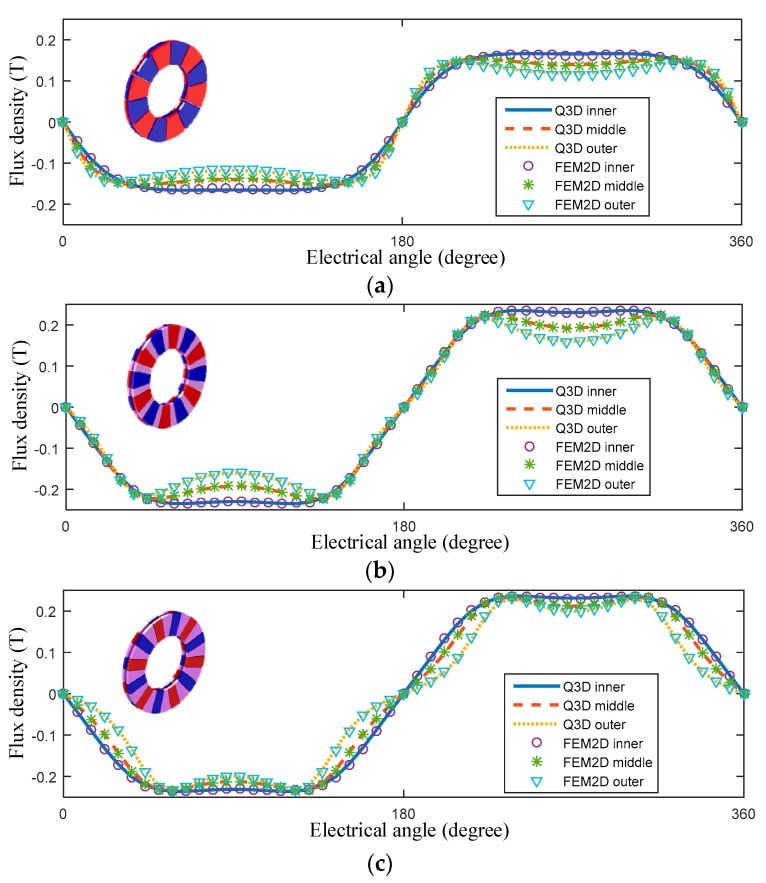

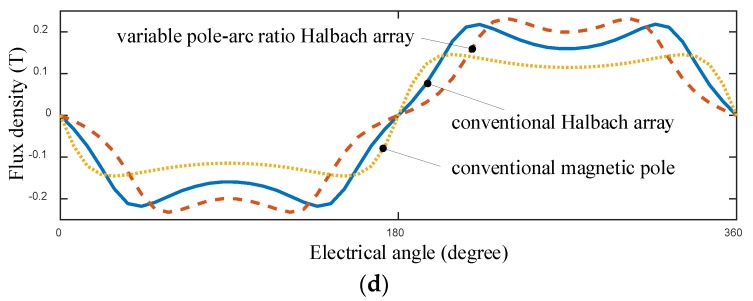
Flux intensity in the air-gap, calculated with quasi-3-D: (**a**–**c**) Comparison between the quasi-3-D and the 2-D finite-element method (FEM) for the conventional magnetic pole, the conventional Halbach array, and VPAR-DHA, respectively; (**d**) Flux intensity at the middle slice.

In order to verify the analytical Quasi-3-D model, the air-gap flux density was compared with the 2-D FEM results. The calculation results in the inner, middle, and outer slices for the conventional magnetic pole, the conventional Halbach array, and the VPAR-DHA are shown in Figure 6. The analytical and 2-D FEM results are in good agreement with each other. As shown in Figure 6d, the flux density distributions at the middle slice are compared for three configurations. Compared with the conventional magnetic pole and the conventional Halbach array, the air-gap magnetic field of the VPAR-DHA is enhanced remarkably. As the pole-arc ratio decreases with the increase of the radius, and the magnetic field enhancement ratio increases with the increase of the radius. Proportional to the radius, the output torque is enhanced significantly with the increase of the outer-ring magnetic field, while the high magnetic field area width decreases at the same time. Because the cross-section width of the coil does not change with the radius, the electrical angle corresponding to the working range decreases with the increase of the radius. The field intensity of the VPAR-DHA can be increased without affecting the working range. 

### 3.2. Correction Function of End-Effects

**Figure 7:**
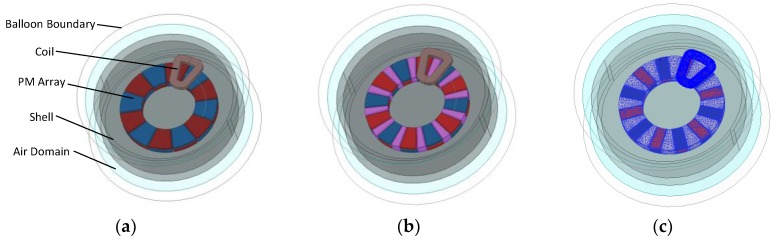
3-D FEM models of different DVCMs: (**a**) Conventional magnetic pole; (**b**) Conventional Halbach array; (**c**) Variable pole-arc ratio Halbach array (with mesh plots).

Obtained by simplifications, the Quasi-3-D method does not take the radial dependence end-effects on the magnetic field into account. For more reliable calculations, proper modeling corrections must be carried out [16,17]. Thus, a 3-D FEM model of the DVCM was built with respect to the analytical results of the quasi-3-D model, with the help of finite-element software ANSOFT. As the DVCM provided in this paper is slotless and back-ironless, the 3-D FEM model was simplified with just the PM array, coils, shell and the air domain. In addition, the balloon boundary was chosen for both fast computation and accounting for the flux leakage effects. As the designed DVCM is an axisymmetric structure, the model was built with a single coil for simplification. The elements were generated with the intelligent tetrahedral meshing tool of ANSOFT, and the total number of elements in the model was up to 200,000. Conventional magnetic pole and conventional Halbach array models were also built for comparison, as shown in Figure 7.

**Figure 8:**
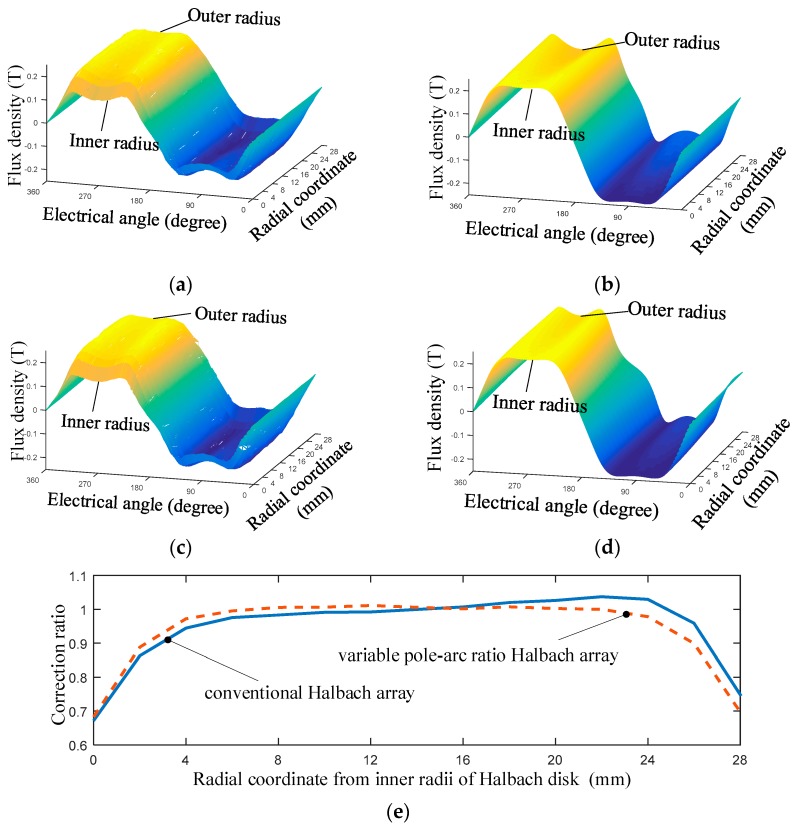
End-effects on the distribution of flux density and the corresponding correction function *g*(*r*): (**a**) 3-D FEM results of conventional Halbach array; (**b**) quasi-3-D results of conventional Halbach array; (**c**) 3-D FEM results of VPAR-DHA; (**d**) quasi-3-D results of VPAR-DHA; (**e**) *g*(*r*).

Comparing quasi-3-D with 3-D FEM, the results in Figure 8a–d show the drop of the flux density near the inner and outer radii. It can be seen that the VPAR-DHA has higher and more stable magnetic field within the working range than the conventional Halbach array, according to both 3-D FEM and quasi-3-D results, but the quasi-3-D results without correction cannot give the accurate description of the end-effects near the inner and outer radii. In this paper, since the exciter only worked in the region with high magnetic field density, the magnetic density ratio between FEM and quasi-3-D results at the center of the working range, where the electric angle is 90°, was used as the correction ratio. The correction function is defined as:

(17) $$g(r_{i})=\frac{B_{FEM}(r_{i},\pi /2)}{B_{Q3D}(r_{i},\pi /2)}$$

The correction functions of the conventional Halbach array and the VPAR-DHA are shown in Figure 8e. The drop can be seen near the inner and the outer radii. Compared with the conventional Halbach array, the correction ratio in the middle region for the VPAR-DHA is closer to 1, which means that the latter has a higher accuracy in analytical calculations.

**Figure 9:**
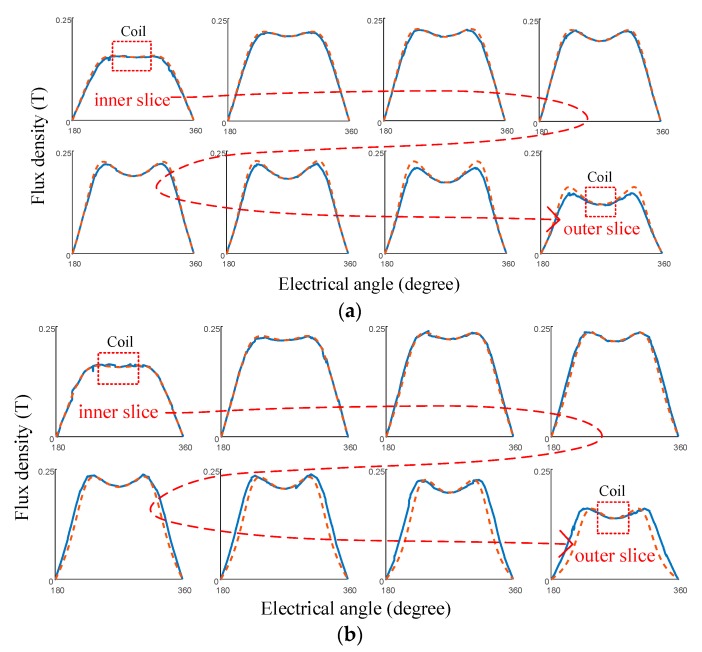
Flux intensity calculated with quasi-3-D and correction functions: (**a**) Conventional Halbach array; (**b**) VPAR-DHA. (Dotted lines and solid lines represent the results of the analytical method and 3-D FEM, respectively).

The results of eight slices calculated with the quasi-3-D models, and the correction function of the conventional Halbach array and VPAR-DHA, are shown in Figure 9. It was found that within the working range of the coil, the end-effect was effectively corrected, and the results are in good agreement with that of 3-D FEM, with errors of less than 5%.

Based on the proposed modeling method of the air-gap field, the torque produced by a current slice at radius $r_{i}$ can be expressed as:

(18) $$T_{slice}(r_{i})=r_{i}A\iint_{s}B(x,y)dxdy$$

where A is the current density, and s is the cross-section of the winding in the working range.

**Figure 10:**
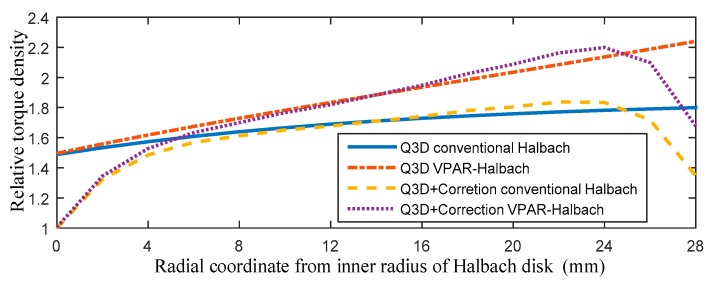
Comparison of relative torque density along the radial coordinates.

Figure 10 shows the relative torque density comparisons, considering the value at the inner radius to be 1. It can be seen that the end-effects weaken the torque output capability near the inner and outer radii. Moreover, compared with the conventional Halbach array, the maximum increase in torque density of the VPAR-DHA at the same radius is 2.2/1.8, and the output torque of the VPAR-DHA is enhanced. It indicates that the proposed VPAR-DHA can effectively increase the output torque capability without changing the rotor inertia.

### 3.3. Design Optimization

With the help of corrected quasi-3-D model, the leading construction and dimensions of the DVCM design can be determined, including the number of phases, coil size, PM volume, outer and inner diameters of the stator and rotor, and air-gap length, etc. A sizing equation of the torque density was proposed for the objective function of the design optimization, as given by:

(19) $$T_{den}=\frac{T_{M}}{J_{M}}=\frac{2\sum_{i=1}^{n}T_{slice}(r_{i})\Delta R}{J_{mag}+J_{y}}=\frac{2\sum_{i=1}^{n}T_{slice}(r_{i})\frac{r_{outer}-r_{inner}}{n}}{\pi \rho_{mag}\left(r_{outer}^{4}-r_{inner}^{4}\right)H_{m}+\frac{1}{2}\pi \rho_{Al}R_{ro}^{4}H_{y}}$$

where $J_{mag}$ and $J_{y}$ are the moments of inertia of the PM array and the aluminum alloy yoke, respectively; n is the number of slices in the quasi-3-D calculation; $\rho_{mag}$ and $\rho_{Al}$ are the density of PM and yoke, respectively; $R_{ro}$ is the rotor radius.

In this design, DVCM was set with the peak power at 2.5 kW and peak output torque at 10 Nm, at a loading frequency of 120 Hz, with a stroke of ±1°; the DVCM diameter is fixed to 220 mm; $H_{y}$ was set to 4 mm, considering the structural strength; the coil width was chosen to be 10 mm with a maximum working range of ±5°. By varying the pole-arc ratio from 0.3 to 0.665, the diameter ratio ($r_{inner}/r_{outer}$) from 0.5 to 0.667, and the PM height from 3 mm to 6 mm, an optimal design of the DVCM structural parameters was obtained by using the objective function with a multi-objective close-form method and the genetic algorithm (GA) method; for details see [19]. The major parameters are listed in Table 1. 

**Figure 11:**
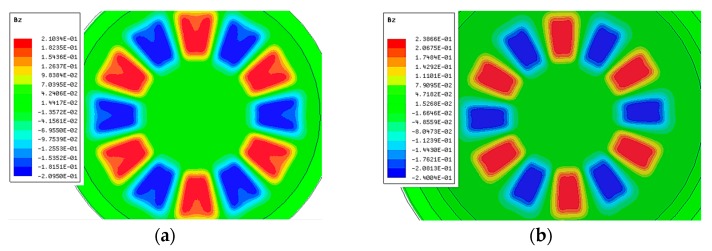
3-D FEM validation of magnetic field optimization: (**a**) Conventional Halbach array; (**b**) Optimization result.

**Figure 12:**
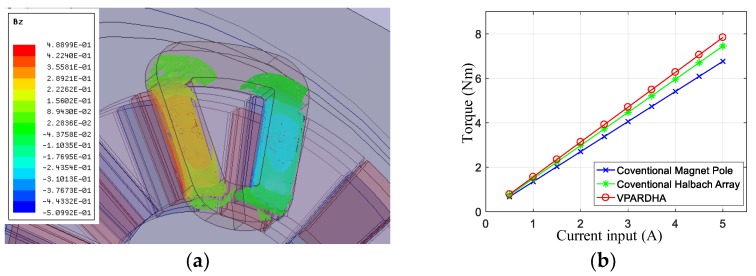
3-D FEM validation with armature reaction field: (**a**) Flux density distribution within the coil; (**b**) Output torque comparison.

The 3-D FEM model in Section 3.2 was used as a virtual prototype to validate the analytical optimization results, as shown in Figure 11 and Figure 12.

Figure 11 shows the optimization results of static magnetic field. The final optimized outer pole-arc ratio of the proposed array was 0.445. Compared with the conventional Halbach array, the max working range of the optimized VPAR-DHA was reduced from ±8° to ±5°. However, the magnetic field fluctuation of the proposed VPAR-DHA was reduced from 10% to less than 1% in the outer side of the array, and the average flux density was promoted by about 10% in the working range.

Figure 12 shows the analysis results with armature reaction field, and a comparison of the output the capability between different types of PM arrays with the same dimensions, except the array structure. The torque constant of a single-coil disk with the proposed Halbach array was 1.54 Nm/A, with good linearity according to the FEM results, which promoted the output torque by at least 7% compared to that in a conventional Halbach array (1.43 Nm/A).

## 4. Prototyping and Experiments

**Figure 13:**
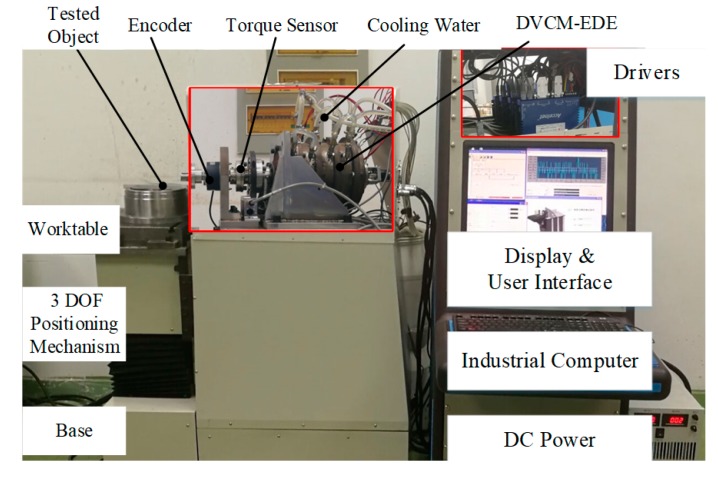
Prototype and test platform.

According to the analysis and the optimization in the previous section, a quad-module DVCM-EDE prototype and its test platform were accomplished, as shown in Figure 13. 

The internal components of the prototype, including the drivers and the loading system, are illustrated in the detailed subfigures for better understanding. The test platform also consists of an industrial computer, driving circuits, feedback sensors, testing sensors, base, 3-DOF positioning system, DC power supply, cooling system, etc.

**Figure 14:**
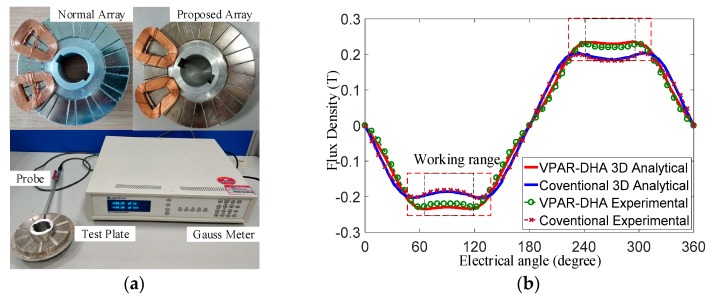
Experiments on flux density: (**a**) Halbach arrays; (**b**) Comparison of the analytical and experimental results.

The proposed VPAR-DHAs used in the prototype were built according to the optimized structural parameters, and the magnetic flux density distribution in the middle slice at a gap length of 4 mm was measured with the Gauss meter Lakeshore 410, as shown in Figure 14a. A conventional Halbach array was also built for comparison. The analytical results and experimental results matched well, with a minor measurement error of less than 5%, as shown in Figure 14b, which proved the validity of the proposed novel magnet array and quasi-3-D modeling method. The flux density within the working range of the proposed array was 10% higher, and more stable than that of the conventional design, which was the same as the FEM analysis results. Minor error was caused by the edge effects of two magnets, assembly error, and measurement error. This would not influence the validity of this design concept. 

**Figure 15:**
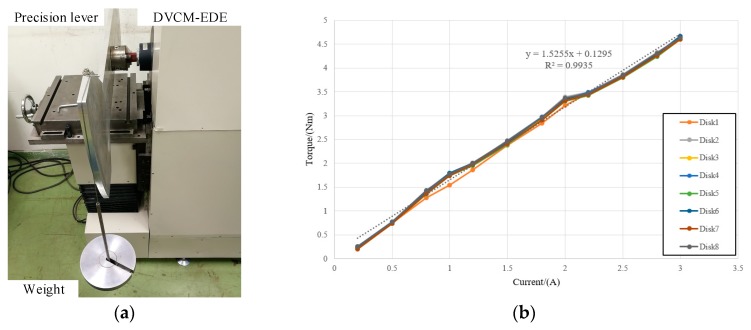
Static experiments with the prototype: (**a**) Lever weight measurement; (**b**) Torque current curve.

In addition, the static output performance was tested with a precision lever and weights when the coils were energized, as shown in Figure 14a. The prototype DVCM-EDE consisted of four DVCMs or eight coil disks. The relationship between the output torque and the input current of each disk is depicted in Figure 15b. The average torque constant of the coil disks was about 1.5255 Nm/A, which fit the FEM results well, and the minimum R^2^ value of the fitting curves was 0.9935. This demonstrated that the torque had an almost linear relation to current, which proved that severe saturation was very small in the magnetic circuit. Thus, stable torque could be achieved, and the controlled model was meanwhile simplified.

**Figure 16:**
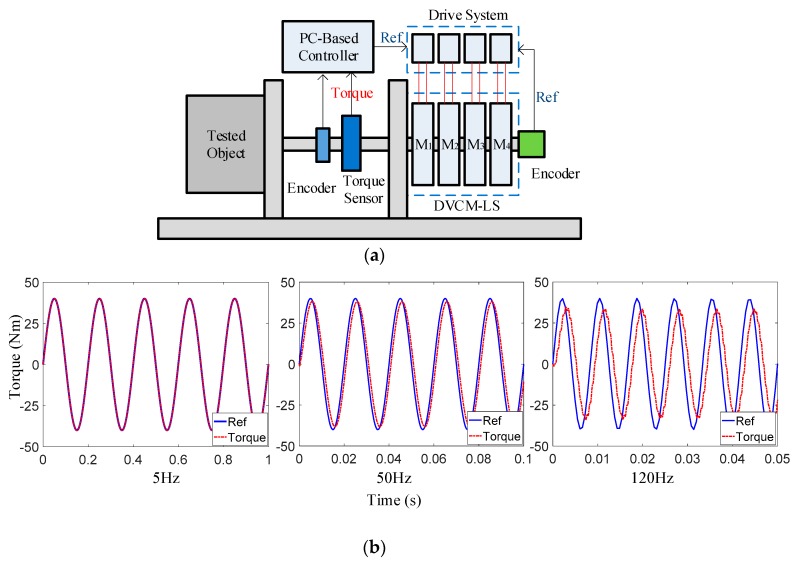
Dynamic experiments with the prototype: (**a**) Operation principle of the prototype; (**b**) Dynamic responses.

The system construct of the prototype is shown in Figure 16a. Each DVCM was driven by an individual DC driver, controlled by a PC-based controller. The exciter worked at two modes: When testing the flexible object, it operated at a feedforward synchronous control method based on the CAN bus, according to its positional reference; while testing rigid objects, it realized the load torque closed-loop synchronous control with the torque sensor. The dynamic tracking responses of the prototype at a representatively low frequency (5 Hz, which is the servo bandwidth of many rotating machinery systems, such as industrial robots), mid-level frequency (50 Hz, which is also near the structural frequency of most rotating machinery systems), and high frequency (120 Hz, which is the performance limitation of the prototype) with a stroke of ±1° and peak dynamic torque 40 Nm, are evaluated in Figure 16b. As the dynamic performances of the prototype gradually changed with the increase of loading frequency, it shows that the DVCM-EDE had a good response of up to 120 Hz, with an amplitude reduction of less than 10%, and a phase lag of less than π/6, which proved the high dynamic characteristics of the proposed exciter in high-frequency applications. For dynamic torsional stiffness test, as the dynamic angular displacement $\theta_{L}$ and the dynamic loading torque $T_{L}$ were directly acquired with the encoder and torque sensor for the calculation of the test results, the small phase lag of $T_{L}$ to reference the signal would not influence the measurement accuracy.

## 5. Conclusions

In this paper, we proposed a VPAR-DHA structure for the design of a multi-module DVCM-EDE used in the high-bandwidth dynamic torsional stiffness test. Compared to the conventional Halbach array, the proposed structure presented a torque promotion of 7% without the increase of rotor inertia. Thus, the dynamic response could be obviously enhanced for high-frequency applications. For fast analysis and the optimization of the electromagnetic structure, a quasi-3-D modeling method was introduced, with end-effects considered by the correction function. Based on the proposed design, prototype experiments were conducted, including flux density and output torque. The DVCM-EDE prototype had a constant torque ratio over its working range, rapid dynamic response, and easy mounting oscillating capability, which proved the effectiveness of this novel structure. Since this paper focuses on the PM array structure optimization, there is still plenty of room to optimize the stator and cooling method. In the future, we will improve the stator structure and the cooling system for a higher performance for the DVCM-EDE, and more convenient assembly. 

## Figures and Tables

**Table 1:** Major Parameters.

Parameter	Symbol	Value
permanent magnet (PM) material	*NdFeB*	N52
Magnetic remanence (T)	$B_{r}$	1.45
DVCM diameter (mm)	$D_{o}$	220
Rotor outer radius (mm)	$R_{ro}$	69
Stator outer radius (mm)	$R_{st}$	83
Pole pairs	*-*	12
Turns per coil	*N*	330
Air gap length (mm)	*G*	1
PM outer radius (mm)	$r_{outer}$	67.5
PM inner radius (mm)	$r_{inner}$	37.5
Pole pitch (degrees)	$\theta_{\tau }$	30
Outer pole-arc ratio	$\alpha_{outer}$	0.445
Inner pole-arc ratio	$\alpha_{inner}$	0.665
Magnet height (mm)	$H_{m}$	4
